# Rupatadine Ameliorates Cisplatin‐Induced Hepatotoxicity in Rats: Targeting HMGB1/TLR4/NF‐κB and NLRP3/Caspase‐1 Signaling Pathways

**DOI:** 10.1002/jbt.71050

**Published:** 2026-07-30

**Authors:** Shaimaa Mohamed Abdelrahman, Amany Abdlrehim Bekhit, Olivia N. Beshay

**Affiliations:** ^1^ Department of Biochemistry, Faculty of Pharmacy Minia University Minia Egypt; ^2^ Department of Biochemistry, Faculty of Pharmacy Minia National University New Minia Egypt

**Keywords:** cisplatin, hepatotoxicity, HMGB1, NLRP3, PAF, rupatadine

## Abstract

Despite cisplatin (CISP) is broadly employed in cancer therapy, its adverse effects, involving hepatotoxicity, restrict its clinical usage. Thus, this study was designed to explore the prospect of repurposing rupatadine (RUPA), a dual antagonist of histamine and platelet‐activating factor (PAF), against CISP‐evoked hepatotoxicity in rats. Rats were *i.p*. injected with CISP (6 mg/kg) for the induction of hepatotoxicity on the 8th day of the study in the presence and absence of RUPA in two dosages (3, 6 mg/kg) orally for 14 days. Different biochemical parameters and histopathological assessments, along with the mechanistic characterizations of the possible protective impact of the RUPA were conducted. Administration of CISP evoked hepatic histopathological modifications and raised serum liver enzyme levels and hepatic MDA content, PAF, and histamine levels, along with diminishing the serum albumin level, hepatic SOD activity, and GSH level. Moreover, CISP remarkably augmented the levels of HMGB1, TLR4, p‐NF‐κB p65, IL‐6, TNF‐α, IL‐18, IL‐1β, NLRP3, and cleaved caspase‐1 proteins with an evident decline in the level of IL‐10. On the contrary, pretreatment with either RUPA.3 or RUPA.6 dramatically ameliorated these alterations triggered by CISP injection. RUPA counteracts hepatotoxicity evoked by CISP in rats via its anti‐oxidative as well as anti‐inflammatory properties via repression of hepatic HMGB1/TLR4/NF‐κB and thus restricts the activation of NLRP3/caspase‐1 signaling cascade.

## Graphical abstract

Graphical representation of the probable mechanisms underlying the effect of RUPA on CISP‐evoked hepatotoxicity in rats.

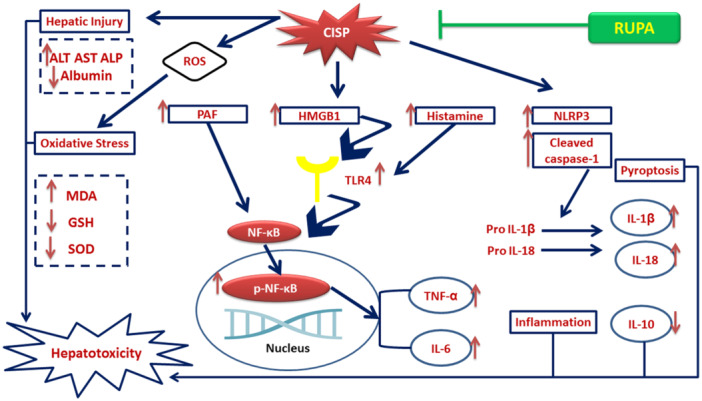

**Abbreviations**
ALP: alkaline phosphatase
ALT: alanine aminotransferase
AST: aspartate aminotransferase
CTRL: control
CMC: carboxymethyl cellulose
CISP: cisplatin
GSH: reduced glutathione
HMGB1: high‐mobility group box 1
*i.p*
: intraperitoneal
IL: interleukin
MDA: malondialdehyde
NLRP3: NOD‐like receptor protein 3
*p*
: probability
PAF: platelet‐activating factor
p‐NF‐κB p65: phosphorylated nuclear factor kappa‐B p65
ROS: reactive oxygen species
RUPA: rupatadine
SOD: superoxide dismutase
TLR4: toll‐like receptor 4
TNF‐α: tumor necrosis factor alpha

## 1 Introduction

Cisplatin (CISP) is one of the most efficient and commonly employed antineoplastic drugs delivered to treat several kinds of tumors, involving testicular, ovarian, head and neck, bladder, lung, cervical cancer, melanoma, and lymphomas [1]. Notwithstanding its efficacy, CISP's clinical applicability is hindered because of its negative effects, especially hepatotoxicity, which may need dose modifications or therapy termination, which compromises therapeutic results [2].

Reactive oxygen species (ROS), generated following CISP administration, trigger oxidative stress, which is among the principal mechanisms that mediate the injury evoked by CISP [3]. A dramatic increase in these harmful free radicals may overcome typical antioxidant defensive systems, triggering significant tissue damage via reacting with macromolecules, which include proteins, membrane lipids, and nucleic acids [4]. Through certain receptors, CISP diffuses into cells. When it builds up inside cancerous and healthy cells, it promotes ROS production and DNA destruction, which yields cell death via necrosis and apoptosis [2].

Importantly, cellular toxic responses like oxidative stress and necrosis boost the release of high mobility group box 1 (HMGB1) [5], which is a ubiquitous protein, exhibiting significance in the pathologic process that includes both acute and chronic liver injury [6, 7]. HMGB1acts on toll‐like receptor 4 (TLR4), activating nuclear factor kappa B (NF‐κB) and promoting the synthesis and expression of several cytokines and pro‐inflammatory mediators involving tumor necrosis factor alpha (TNF‐α) and interleukin (IL)‐6, contributing to progressive tissue injury [8].

Pyroptosis, a kind of cell death modulated by inflammasome induction, was previously reported as a significant contributor to hepatocyte injury and hepatic inflammation [9, 10]. Notably, oxidative stress as well as TLR4/NF‐κB up‐regulate and activate the nucleotide‐binding oligomerization domain (NOD)‐like receptor family pyrin domain containing 3 (NLRP3) inflammasome. NLRP3 activation, resulting in caspase‐1‐modulated cleavage of proinflammatory cytokines, including IL‐1β and IL‐18, into their mature active forms [11].

Platelet‐activating factor (PAF) is a ubiquitous phospholipid mediator and has been included in a broad range of pathological states, like hepatotoxicity [12]. Remarkably, growing evidence revealed that PAF is involved in the pathologic process of hepatotoxicity evoked by the chemotherapeutic drug 5‐fluorouracil [13].

Rupatadine (RUPA), a member of the second generation of antihistamines, possesses dual affinity for blocking both histamine‐1 and PAF receptors; it is widely utilized for the management of chronic urticaria and allergic disorders [14]. Numerous research studies have investigated that RUPA exhibits remarkable antioxidant and anti‐inflammatory properties [15, 16, 17]. Recently, it was reported as a novel agent for treating colorectal cancer [18]. Moreover, it was documented that RUPA has anti‐fibrotic potential against diethylnitrosamine‐evoked hepatic fibrosis [19] and bleomycin as well as silica‐induced pulmonary fibrosis [20]. However, new functional and mechanistic actions of RUPA against CISP‐induced liver toxicity are yet to be identified.

In accordance with previous findings, along with drug repurposing strategies, testing for new therapeutic agents may offer considerable promise in providing beneficial effects to different illnesses [21, 22]. Thus, this research is designed to explore the probable protective role of RUPA against hepatotoxicity evoked by CISP and to explore the underlying mechanisms of RUPA's effect with a focus on oxidative stress and inflammation, along with its modulatory action on HMGB1/TLR4/NF‐κB as well as NLRP3/caspase‐1/IL‐1β signaling cascades.

## 2 Materials and Methods

### 2.1 Drugs

RUPA was sourced from Mash Premiere for Pharmaceutical Industries (Cairo, Egypt) and suspended (0.5 percentage w/v) in carboxy methylcellulose (CMC) solution. CISP was procured from Mylan Institutional LLC (Rockford, USA).

### 2.2 Experimental Animals

This investigation involved forty adult male Wistar albino rats with ages approximately from 8 to 10 weeks and a weight of 200 to 220 g; they were procured from the National Research Center (Giza, Egypt). Rats underwent a 14‐day habituation period in the lab prior to the commencement of the study protocol. Throughout the experiment, rats received unrestricted access to food alongside water ad libitum. All animal testing experiments comply with the ARRIVE guidelines. Our experimental protocol was reviewed and approved by the Research Ethics Committee for the Care and Use of Laboratory Animals, Faculty of Pharmacy, Minia University, Egypt (approval code: MPEC (250505)) in accordance with EU Directive 2010/63 for the protection of animals used for scientific purposes.

### 2.3 Study Protocol

Rats were distributed at random into 5 groups (8 rats/group) to evaluate the possible hepatoprotective impact of RUPA on CISP‐evoked hepatic toxicity (Figure 1). In Group I (control (CTRL)), rats received 0.5% CMC orally for 14 successive days and a single intraperitoneal (*i.p*.) injection of saline solution on day 8. In Group II (RUPA.6), rats were pretreated with RUPA at a dose of 6 mg/kg orally [23] for 14 successive days. Group III (CISP) rats received 0.5% CMC orally for 14 successive days along with a single injection of 6 mg/kg CISP *i.p* [24, 25]. on day 8. Rats in Group IV (RUPA.3 + CISP) and Group V (RUPA.6 + CISP) received 3 and 6 mg/kg RUPA, respectively, orally [23] for 14 successive days along with a one injection of 6 mg/kg CISP *i.p*. on day 8.

**Figure 1:**
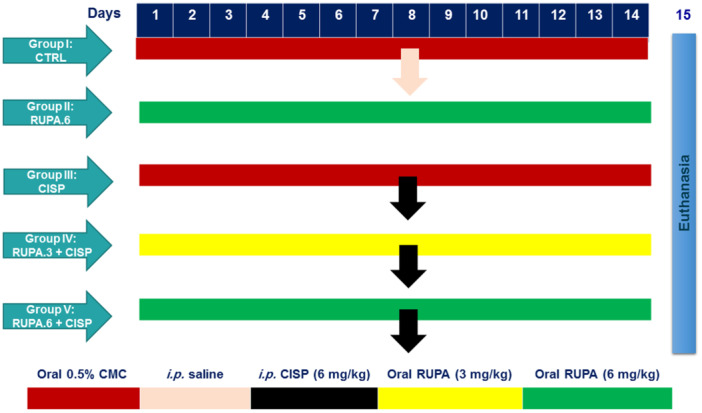
Schematic drawing of the study timeline.

### 2.4 Tissue Isolation and Specimen Preparation

On the 15th day of the investigation's completion, animals were anesthetized with isoflurane 5%. Before euthanasia by decapitation, blood was quickly drawn from the jugular vein to separate the serum. Next, serum specimens were screened for different biochemical parameters. The liver from each animal was promptly excised, gently washed with cold phosphate‐buffered saline, and divided into 3 portions: the first portion was kept in 10% formalin for histopathology, along with immunohistochemical investigations. The second portion was homogenized in cold PBS (0.05 M, pH 7.4) and followed by centrifugation to isolate the supernatant for estimating the oxidative stress biomarkers. While the third portion was maintained at −80°C until further biological molecular assays.

### 2.5 Determination of Serum Biomarkers

Levels of ALT, AST, and ALP were assessed in the serum employing commercial kits (Cat. No: GPT113100, GOT111060, and ALP101090, respectively) from BioMed Diagnostics (Badr City, Egypt). Moreover, serum albumin level was detected with the kit (Cat. No: 210001) from Spectrum Diagnostics (Cairo, Egypt). Measurements were accomplished firmly following the kit's instructions.

### 2.6 Histopathology Evaluation

Part of every liver tissue was stored in 10% neutral formalin saline, then processed to obtain paraffin blocks. Five‐μm liver sections were subjected to cutting employing a rotatory microtome, mounted on glass slides, and treated with hematoxylin and eosin (H&E) stain. H&E‐stained liver slides were visualized and photographed utilizing an Olympus BX50 microscope connected with a digital camera (Tokyo, Japan).

### 2.7 Immunohistochemical Protein Assay

Another five μm‐thick hepatic sections were placed on positively charged slides and processed for immunohistochemical staining with streptavidin‐biotin amplifying system. The sections were then incubated overnight at 4°C with primary antibody against phosphorylated (p)‐NF‐κB p65 (Cat. No. ES1368, ELK Biotechnology, China). Afterward, secondary antibody was applied at room temperature for 30 min, prior to the addition of 3,3′‐diaminobenzidine tetrahydrochloride reagent. Following the washing of the 3,3′‐diaminobenzidine tetrahydrochloride reagent, slides were hematoxylin counterstained and covered. % positive area of immunohistochemical expression of p‐NF‐κB p65 by utilizing Fiji Image J software was basically used for this assessment [26].

### 2.8 Determination of Oxidant/Antioxidant Biomarkers

The hepatic content of malondialdehyde (MDA) (Cat No. MD 25 29), reduced glutathione (GSH) (Cat No. GR 25 11), in addition to superoxide dismutase (SOD) (Cat No. SD 25 21), was estimated in the liver tissue homogenate, as guided by commercial kits supplied from Biodiagnostics Co. (Giza, Egypt).

### 2.9 ELISA Technique

ELISA kits (MyBioSource Inc., California, USA) were utilized to evaluate PAF (Cat. No: MBS722041), histamine (Cat. No: MBS732202), HMGB‐1(Cat. No: MBS703437), and IL‐18 (Cat. No: MBS355269) in hepatic homogenates. Moreover, biochemical evaluations of TLR4, IL‐6, TNF‐α, IL‐1β, and IL‐10 in the liver homogenates were carried out utilizing ELISA kits (Cat. No: SEA753Ra, SEA079Ra, SEA133Ra, SEA563Ra, and SEA056Ra, respectively) from Cloud Clone Corp. (Texas, USA). The assays followed the manufacturer's suggested guidelines.

### 2.10 Western Blotting

The NLRP3 and cleaved caspase‐1 proteins were detected in the hepatic homogenate by applying immunoblotting. Briefly, the ReadyPrepTM protein extraction kit sourced from Bio‐Rad Inc. (Cat. No: 163‐2086) was employed in conformity with supplier guidelines in order to extract total protein in homogenized liver specimens. Following quantifying total protein in every specimen via Bradford assay [27], 20 μg of the protein specimens were loaded and separated employing SDS‐PAGE. Protein specimens were blotted to polyvinylidene fluoride membranes following the electrophoresis process. Membranes were then put in a blocking solution at room temperature for 1 h before being probed with antibodies against NLRP3 (Cat. No: ab263899, Abcam, Cambridge, UK), cleaved caspase‐1 (Cat. No: PA5‐105049, Thermo Fisher Scientific, USA), and β‐actin (Cat. No: ab8226, Abcam, Cambridge, UK) overnight at 4°C. The primary antibodies were removed by washing numerous times, preceding pretreatment with horseradish peroxidase‐conjugated secondary antibody solution for 1 h at ambient temperature. Eventually, by utilizing an enhanced chemiluminescence kit (Clarity TM Western ECL substrate) provided by Bio‐Rad with Cat. No: 170–5060 in line with the supplier's suggested guidelines, the protein bands were detected, and a CCD camera‐based imager was utilized for capturing the chemiluminescent signals. The target protein band values were quantified in relation to the housekeeping protein β‐actin, utilizing TotalLab analysis software (Version 1.0.1, www.totallab.com), and data were displayed as a fold change related to the CTRL group.

### 2.11 Statistical Analysis

GraphPad Prism version 9.00 (San Diego, CA) was applied to achieve statistical analyses as well as graphs. Experimental values were represented as mean ± SEM. One‐way ANOVA test was implemented for comparisons among different groups, followed by Tukey's post hoc test. A probability (*p*) value < 0.05 was identified for all analyses as a criterion for statistical significance.

## 3 Results

### 3.1 RUPA Attenuated CISP‐Evoked Hepatotoxicity

Serum ALT, AST, ALP, and albumin levels were evaluated to appraise the positive impact of RUPA on CISP‐evoked hepatotoxicity. Related to the normal CTRL group, CISP injection exhibited a remarkable elevation in serum ALT, AST, and ALP levels. Furthermore, it culminated in a notable decline in the albumin level in the serum. Conversely, RUPA pretreatment at a dosage of 3 or 6 mg kg^− 1^ effectively reversed these changes, yielding a profound reduction in serum ALT, AST, and ALP levels alongside a considerable elevation in albumin level as contrasted to the CISP group. Interestingly, pretreatment with RUPA.6 exhibited a significant change in ALT, AST, ALP, and albumin serum levels in contrast to the RUPA.3 group (Table 1).

**Table 1:** Effect of RUPA administration on serum ALT, AST, ALP, and albumin of CISP‐treated rats.

Groups	ALT (U/L)	AST (U/L)	ALP (U/L)	Albumin (g/dL)
CTRL	32.04 ± 2.101	107.0 ± 4.085	109.1 ± 7.850	4.198 ± 0.087
RUPA.6	34.49 ± 2.989	113.9 ± 5.124	110.4 ± 11.37	4.186 ± 0.090
CISP	109.2 ± 7.491^a^	237.2 ± 18.83^a^	246.1 ± 14.41^a^	2.964 ± 0.079^a^
RUPA.3 + CISP	70.98 ± 4.762^a,b^	170.0 ± 10.61^a,b^	179.3 ± 11.45^a,b^	3.458 ± 0.093^a,b^
RUPA.6 + CISP	44.95 ± 5.390^b,c^	125.5 ± 7.612^b,c^	128.9 ± 9.158^b,c^	3.943 ± 0.177^b,c^

*Note:* Values are represented as mean ± SEM (*n* = 8/group). Multiple comparisons were carried out applying One‐way ANOVA followed by Tukey's post‐hoc test. ^a, b, and c^: Significantly (*p* < 0.05) different compared to CTRL, CISP, and RUPA.3 + CISP groups, respectively.

### 3.2 RUPA Ameliorated CISP‐Evoked Liver Histopathological Changes

Hepatic sections from CTRL and RUPA.6 groups showed normal histological structure of hepatic lobules formed of a central vein surrounded by normal hepatocytes arranged around sinusoids. Conversely, examination of the CISP group demonstrated congested central vein and sinusoids, necrotized hepatocytes with pyknotic bodies, and inflammatory cells. In the RUPA.3 + CISP group, dilated congested central vein and sinusoids, hepatocytes with cytoplasmic vacuolations, and a few pyknotic bodies were observed. Pretreatment with RUPA.6 to the CISP‐intoxicated group showed mild congestion of central vein and sinusoids and a few hepatocytes with cytoplasmic vacuolations (Figure 2).

**Figure 2:**
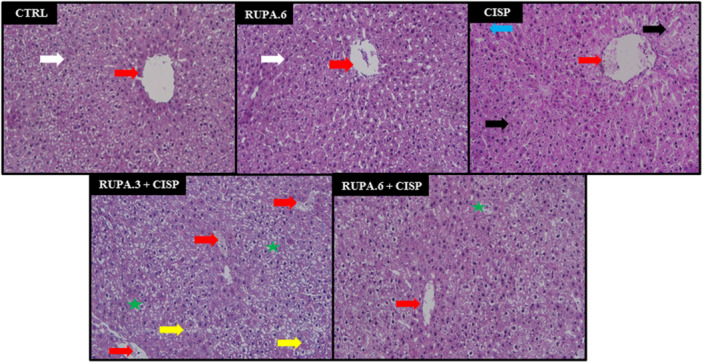
Effect of RUPA on pathological alterations of CISP‐injected rats. Representative pictures of H&E‐stained hepatic sections from CTRL and RUPA.6 treated animals showing normal hepatic lobules formed of central vein (red arrows) surrounded by normal hepatocytes (white arrows) arranged around sinusoids. CISP‐injected animals showing congested central vein and sinusoids (red arrow), necrotized hepatocytes with pyknotic bodies (black arrow), and inflammatory cells (blue arrow). RUPA.3‐pretreated animals showing dilated congested central vein and sinusoids (red arrow), hepatocytes with cytoplasmic vacuolations (green star), and few pyknotic bodies (yellow arrow). RUPA.6‐pretreated animals showing mild congestion of central vein and sinusoids (red arrow) and few hepatocytes with cytoplasmic vacuolations (green star) (H&E X200, scale bar = 50 µm).

### 3.3 RUPA Mitigated CISP‐Evoked Oxidative Stress

The influence of RUPA on the hepatic redox alterations was investigated by assessing the antioxidant status in addition to the MDA content. Relative to the CTRL group, injection of CISP caused a remarkable reduction in GSH level and SOD activity alongside a considerable elevation in MDA content. Meanwhile, pretreatment of rats with RUPA (3 or 6 mg kg^−1^) notably elevated GSH level and SOD activity as well as markedly suppressed MDA content relative to rats injected with CISP. Notably, a remarkable distinction did emerge between the pretreatment with the lower and higher dosages of RUPA for all oxidative stress parameters (Table 2).

**Table 2:** Effect of RUPA administration on hepatic oxidative stress biomarkers: MDA content, GSH level, and SOD activity of CISP‐treated rats.

Groups	MDA (nmol/gm tissue)	GSH (mg/gm tissue)	SOD (U/gm tissue)
CTRL	83.14 ± 2.914	4.396 ± 0.141	959.3 ± 36.14
RUPA.6	76.80 ± 3.036	4.599 ± 0.120	942.8 ± 26.97
CISP	156.9 ± 7.391^a^	1.834 ± 0.144^a^	409.3 ± 42.96^a^
RUPA.3 + CISP	118.3 ± 6.572^a,b^	2.879 ± 0.202^a,b^	606.7 ± 37.11^a,b^
RUPA.6 + CISP	96.71 ± 4.679^b,c^	3.705 ± 0.217^a,b,c^	823.3 ± 65.37^b,c^

*Note:* Values are represented as mean ± SEM (*n* = 8/group). Multiple comparisons were carried out applying One‐way ANOVA followed by Tukey's post‐hoc test. ^a, b, and c^: Significantly (*p* < 0.05) different compared to CTRL, CISP, and RUPA.3 + CISP groups, respectively.

### 3.4 Effect of RUPA on Hepatic PAF and Histamine Levels

The CISP‐injected rats exhibited a pronounced rise in both levels of PAF (Figure 3A) and histamine (Figure 3B) in comparison with the unchallenged CTRL rats. On the contrary, RUPA administration at a dosage of 3 or 6 mg kg^−1^ triggered a substantial reduction in the levels of PAF and histamine in contrast to the CISP group. Remarkably, RUPA's high dose showed a considerable reduction in PAF and histamine levels in comparison to the lower dose.

**Figure 3:**
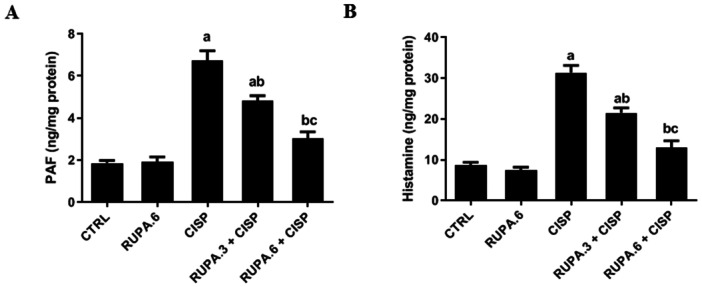
Effect of RUPA on hepatic PAF (A) and histamine (B) levels of CISP‐injected rats. Values are represented as mean ± SEM (*n* = 8/group). Multiple comparisons were carried out applying One‐way ANOVA followed by Tukey's post‐hoc test. ^a, b, and c^: Significantly (*p* < 0.05) different compared to CTRL, CISP, and RUPA.3 + CISP groups, respectively.

### 3.5 RUPA Dampened CISP‐Evoked Inflammatory Burden

#### 3.5.1 Effect on the Hepatic Level of HMGB1 and TLR4 Proteins

CISP‐injected rats exhibited a remarkable augmentation in the level of HMGB1 and TLR4 proteins relative to the normal CTRL group. On the contrary, RUPA‐treated groups exhibited a notable decline in the level of HMGB1 and TLR4 proteins in contrast to the CISP‐injured group. Of interest, the decrease in HMGB1 as well as TLR4 protein levels displayed a heightened prominence in CISP rats which were pretreated with a 6 mg kg^‐1^ dosage of RUPA, as compared with those receiving a 3 mg kg^‐1^ dose, as depicted in (Figure 4A,B).

**Figure 4:**
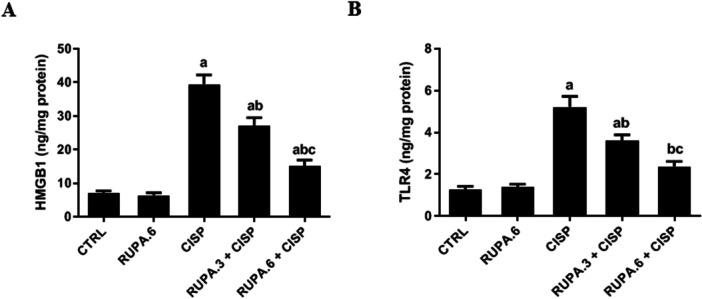
Effect of RUPA on hepatic HMGB1 (A) and TLR4 (B) levels of CISP‐injected rats. Values are represented as mean ± SEM (*n* = 8/group). Multiple comparisons were carried out applying One‐way ANOVA followed by Tukey's post‐hoc test. ^a, b, and c^: Significantly (*p* < 0.05) different compared to CTRL, CISP, and RUPA.3 + CISP groups, respectively.

#### 3.5.2 Effect on the Hepatic Expression of p‐NF‐κB p65

Immunohistochemical staining of p‐NF‐κBp 65 sections of hepatic tissue in both CTRL and RUPA.6 groups showed negative immune‐expression of p‐NF‐κB p65. On the contrary, the CISP group showed strongly positive immune‐expression of p‐NF‐κB p65. CISP‐intoxicated group pretreated with RUPA.3 showed weak positive expression of p‐NF‐κB p65. Interestingly, RUPA.6 pretreatment to the CISP‐intoxicated group resulted in a negative immune‐expression of p‐NF‐κB p65 (Figure 5A).

**Figure 5:**
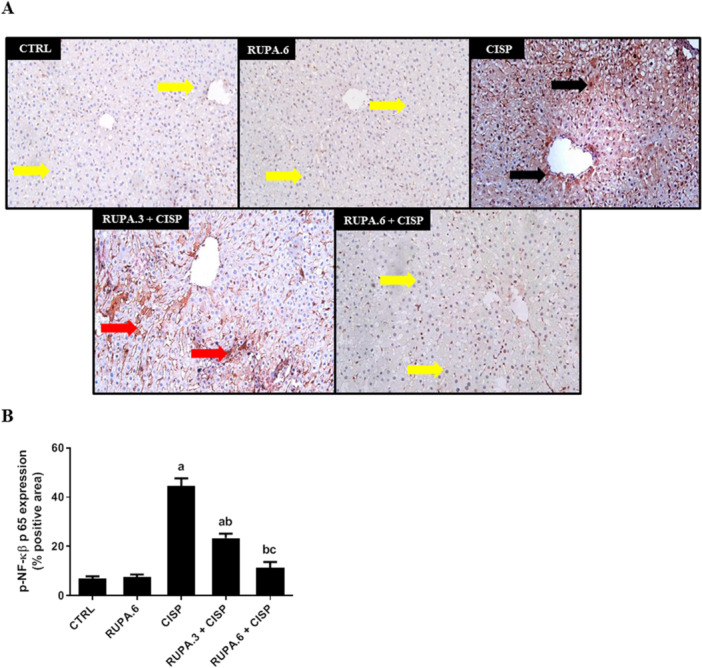
Representative photomicrographs of immunohistochemical staining showing hepatic tissue expression of p‐NF‐κB p65 (A) in different studied groups. p‐NF‐κB p65 staining of hepatic sections of CTRL, RUPA.6, and RUPA.6 + CISP groups showing negative immune‐expression (yellow arrows). CISP group showing strongly positive immune‐expression of p‐NF‐κB p65 (black arrows). Pretreated CISP group with RUPA.3 showing weak positive immune‐expression of p‐NF‐κBp65 (red arrows). (Immunohistochemistry by p‐NF‐κB p65 antibody, X200, scale bar = 50 µm). % positive area of immunohistochemical expression of p‐NF‐κB p65 (B). Values are represented as mean ± SEM (n = 8/group). Multiple comparisons were carried out applying One‐way ANOVA followed by Tukey's post‐hoc test. ^a, b, and c^: Significantly (*p* < 0.05) different compared to CTRL, CISP, and RUPA.3 + CISP groups, respectively.

Results of semi‐quantitative analysis showed a considerable elevation of the % positive area of immunohistochemical expression of p‐NF‐κB p65 in the CISP group in comparison with the CTRL group. Conversely, pretreatment of RUPA.3 or RUPA.6 showed a remarkable reduction in the percentage positive area of immunohistochemical expression of p‐NF‐κB p65 in contrast to the CISP group. In addition, the percentage of positive area of immunohistochemical expression of p‐NF‐κB p65 was substantially decreased in the RUPA.6 + CISP group relative to the RUPA.3 + CISP group (Figure 5B).

#### 3.5.3 Effect on Pro‐Inflammatory and Anti‐Inflammatory Mediators

Considering the protective impact of RUPA on the hepatic tissues at both the biochemical and histological levels, we confirm our findings by investigating the influence of RUPA on mediating liver tissue inflammation. Consequently, levels of IL‐6, TNF‐α, IL‐1β, IL‐18, and IL‐10 were assessed in the hepatic tissues of CISP‐treated rats with and without pretreatment utilizing the ELISA technique. Rats injected with CISP revealed a noticeable elevation in the hepatic protein levels of IL‐6 (Figure 6A), TNF‐α (Figure 6B), IL‐18 (Figure 6C), and IL‐1β (Figure 6D), indicating the inflammatory response evoked by CISP in the liver as compared to the CTRL rats. Additionally, it exhibited a remarkable decline in IL‐10 (Figure 6E) protein level. In contrast, when rats were treated with RUPA.3 or RUPA.6, the hepatic levels of TNF‐α, IL‐18, IL‐6, and IL‐1β proteins markedly reduced with a concurrent elevation in the hepatic protein level of IL‐10 as compared to CISP‐injected rats. It is noteworthy that the high dose of RUPA dramatically suppressed the hepatic levels of pro‐inflammatory mediators, alongside substantially elevating the anti‐inflammatory IL‐10 in comparison with the low dose. Overall, this data suggests that RUPA modulated hepatic tissue inflammation evoked by CISP injection via diminishing the proinflammatory biomarkers, simultaneously restoring the level of the key anti‐inflammatory protein.

**Figure 6:**
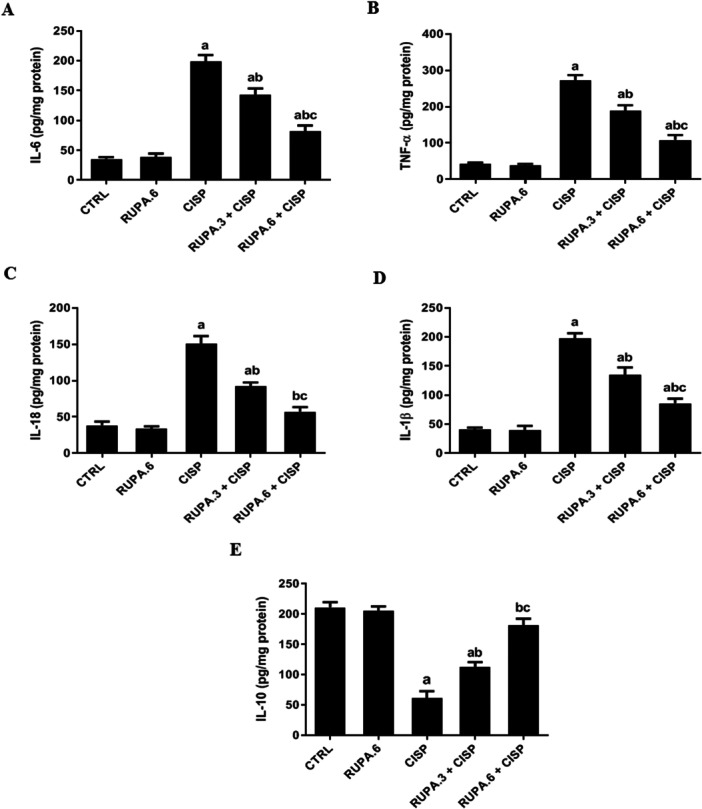
Effect of RUPA on hepatic IL‐6 (A), TNF‐α (B), IL‐18 (C), IL‐1β (D) and IL‐10 (E) of CISP‐injected rats. Values are represented as mean ± SEM (*n* = 8/group). Multiple comparisons were carried out applying One‐way ANOVA followed by Tukey's post‐hoc test. ^a, b, and c^: Significantly (*p* < 0.05) different compared to CTRL, CISP, and RUPA.3 + CISP groups, respectively.

### 3.6 RUPA Suppressed CISP‐Evoked Hepatic Pyroptosis

As depicted in Figure 7, CISP intoxication greatly augmented the expression of NLRP3 as well as cleaved caspase‐1 proteins in the liver relative to the CTRL group. Conversely, RUPA, when administered to rats at either a low or high dose with CISP, considerably inhibited the elevation of NLRP3 and cleaved caspase‐1 relative to CISP‐injected rats. Intriguingly, the anti‐pyroptosis action of RUPA at a 6 mg/kg dosage was remarkably more robust in contrast to the effect of 3 mg/kg RUPA pretreatment in CISP‐intoxicated rats. In sum, these findings suggested a hepatoprotective effect of RUPA via suppressing the triggering of the NLRP3 inflammasome and consequently the pyroptosis impact.

**Figure 7:**
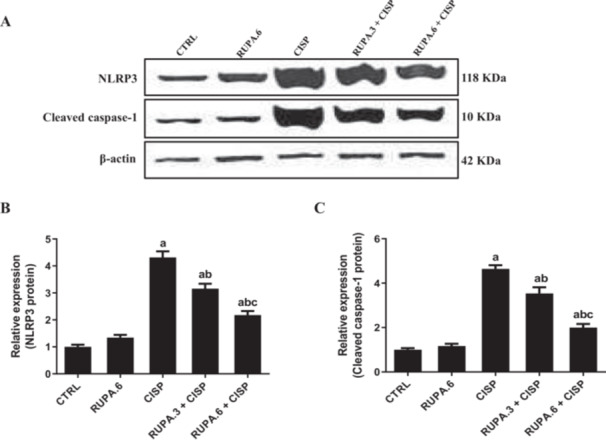
Effect of RUPA on the expression of NLRP3 and cleaved caspase‐1 proteins of CISP‐injected rats. (A) Representative immunoblots depicting the NLRP3, cleaved caspase‐1, and β‐actin proteins across different experimental groups. (B and C) Quantitative analysis of NLRP3 and cleaved caspase‐1 proteins expression after normalization using internal reference β‐actin, and levels of expression were graphed as a fold change in reference to CTRL group. Values are represented as mean ± SEM (*n* = 3/group). Multiple comparisons were carried out applying One‐way ANOVA followed by Tukey's post‐hoc test. ^a, b, and c^: Significantly (*p* < 0.05) different compared to CTRL, CISP, and RUPA.3 + CISP groups, respectively.

## 4 Discussion

The Food and Drug Administration authorized CISP, a strong platinum‐derived antineoplastic agent, in 1978 [28]. Although it has positive effects on solid cancers, CISP‐induced toxic impact in a variety of tissues, including the kidney, liver, and heart, hinders its therapeutic applications [28, 29]. Thus, approaches for incorporating CISP plus other agents, either natural [30] or synthetic [31], are critical for alleviating CISP‐evoked hepatotoxicity. Accordingly, the present research's purpose is to inspect the potential repurposing of RUPA for attenuating CISP‐evoked hepatotoxicity and to clarify the possible modulatory effect of RUPA on HMGB1/TLR4/NF‐κB, along with NLRP3/caspase‐1/IL‐1β signaling cascades.

In this study, an increase in serum ALT, AST, and ALP levels, along with a reduction in albumin levels, indicated that liver injury was initiated by CISP administration. These findings corroborate earlier studies that reported similar alterations in the levels of previous markers triggered by CISP injection [32, 33]. Additionally, histopathological changes were identified, reflecting the biochemical findings. Furthermore, all of the evaluated biomarkers and the hepatic histopathological changes were improved in CISP‐injected rats when pretreated with RUPA. This suggests that RUPA protects the liver from damage caused by CISP.

Accordingly, the results paved an avenue for us to conduct additional assessments for clarifying the molecular bases of RUPA's hepatoprotective actions against CISP‐evoked hepatotoxicity. We evaluated the oxidative stress status in CISP‐intoxicated rats in the absence and presence of RUPA pretreatment, considering that oxidative stress is a principal mechanism in the pathologic process of hepatic injury triggered by CISP injection. It is well‐established that the generation of ROS is elevated after CISP administration [34], terminating in affecting cells in a direct manner by promoting various changes in their structure along with function [35]. Consequently, the cell's antioxidant defense mechanism is essential for combating oxidative stress, along with shielding the cell from the probably detrimental implications of ROS and oxidative damage [36].

Our study identified that CISP‐treated rats exhibited increased oxidative burden status and diminished antioxidant defense, as reflected by elevated MDA level as a lipid peroxidation indicator, plus reduction in antioxidant status, GSH level, and SOD activity. Many studies supported the outcomes of this investigation [30, 37, 38]. On the other hand, pretreatment of CISP‐injected rats with RUPA revealed obvious antioxidant action, including GSH levels and SOD activities in the hepatic tissues, which were raised, and MDA contents were diminished, identifying RUPA's antioxidant potential, which is consistent with Khalaf et al. [13].

In addition to the oxidative stress, inflammation is another pathogenic critical mechanism that modulates CISP‐evoked hepatotoxicity [39]. The HMGB1/TLR4/NF‐kB trajectory is a well‐recognized inflammatory pathway in which ROS stimulates HMGB1, which binds to TLR4 [40]. In fact, the triggering of the HMGB1‐TLR4 axis promotes upregulation of the p‐NF‐kB [41]. The latter enhanced the generation of the inflammatory markers involving TNF‐α and IL‐6 [42]. This study identified that CISP injection can activate the HMGB1/TLR4/NF‐kB signaling cascade, which was confirmed by assessing the HMGB1, TLR4, and p‐NF‐kBp65 protein expression of CISP‐administered rats in comparison to the CTRL group rats. We identified upregulation of all protein expression in hepatic tissues of CISP‐injected rats. These conclusions are in harmony with Man et al. [43] and Hassan et al. [44], who stated that CISP injection elevated the protein expression of HMGB1in the hepatic tissues. Additionally, Malheiro et al. [45] revealed that CISP‐injected rats exhibited increased expression of TLR4 and NF‐kB genes, as well as elevated NF‐kBp65 protein levels in hepatic tissues, relative to the CTRL rats.

RUPA pretreatment in CISP‐administered rats modulated the hepatic HMGB1/TLR4/NF‐kB signaling axis by downregulating HMGB1, TLR4, as well as p‐NF‐kB p65 protein expression relative to CISP‐intoxicated untreated rats. In agreement with our study, the level of HMGB1 protein following RUPA treatment in the diethylnitrosamine‐evoked liver fibrosis model of rats was demonstrated [16]. Additionally, our results were consistent with a recent study, which established that RUPA administration attenuated the inflammatory reaction in hepatic tissues of lipopolysaccharide‐intoxicated rats via inhibiting TLR4 and NF‐κβ p65 protein expression [46].

Following the detection of the significance of hepatic HMGB1/TLR4/NF‐kB expression modulation by RUPA pretreatment, we also evaluated the expression of different pro‐inflammatory biomarkers. Hepatic tissue levels of pro‐inflammatory biomarkers like IL‐6, TNF‐α, IL‐18, as well as IL‐1β were increased in CISP‐injected rats. Our results are aligned with Rashid et al. [47], who established that TNF‐α, IL‐1β plus IL‐6 levels were raised in the group intoxicated with CISP relative to the CTRL group. Furthermore, IL‐10 has been implicated in many investigations as a cytokine that protects against hepatic injury [48, 49]. In our study, a reduction in hepatic IL‐10 was identified in the CISP group, which is consistent with earlier studies [47, 50].

Pretreatment with RUPA suppressed the inflammatory response, as confirmed by reduced protein levels of IL‐6, TNF‐α, IL‐18, and IL‐1β, while maintaining IL‐10 levels, revealing the anti‐inflammatory action of RUPA. In parallel with our findings, it was reported that RUPA diminished the level of pro‐inflammatory biomarkers (TNF‐α, IL‐6, and IL‐1β) in the hepatic tissues of 5‐fluorouracil‐intoxicated rats [13]. Moreover, it reduced the previously mentioned inflammatory mediators alongside raising the level of IL‐10 as an anti‐inflammatory cytokine in the intestine of 5‐fluorouracil‐intoxicated rats [51].

One of the crucial signaling pathways engaged in hepatic injury pathogenesis is the NLRP3 inflammasome [52]. ROS, a well‐recognized activator of the NLRP3 inflammasome, acts as a fundamental aspect in promoting the NLRP3 inflammasome activation in response to various exogenous stimuli in addition to endogenously secreted molecules from damaged cells [53]. Following its activation, it cleaves procaspase‐1 into its active form via autocatalysis. Then, the activated form of caspase‐1 converts pro‐IL‐1β as well as pro‐IL‐18 to their mature forms, IL‐1β and IL‐18, respectively. This mechanism exhibits a beneficial impact at the earliest stage of inflammation [54]. However, when IL‐1β and IL‐18 levels are prolonged, generated, and built up within the cell, they trigger pyroptosis, tissue destruction, and organ dysfunction [55].

In the current work, NLRP3 and cleaved caspase‐1 protein expression were elevated in the CISP‐evoked hepatotoxicity rat model. These findings, considering NLRP3 activation following CISP administration, were identified to be in the same line as Qu et al. [56]. Thus, RUPA pretreatment was shown to alleviate pathogenic alteration via suppressing the triggering of NLRP3 inflammasome and the consequent inflammatory mediators.

PAF is a strong proinflammatory phospholipid, released by many types of cells, which mediates a variety of pathological and physiological actions, such as wound healing, inflammation, apoptosis, and angiogenesis [57]. Pavão dos Santos et al. [58] reported that BN 52063, a PAF antagonist, exhibited a protective action against CISP‐induced nephrotoxicity, resulting in approximately normalisation of all markers of kidney function. Additionally, several studies demonstrated that antihistaminic drugs showed nephroprotective action against kidney toxicity triggered by CISP injection [59, 60]. Moreover, a previous study proved that endogenous histamine acting on H1 receptors is regarded as an exacerbating mediator that triggers the development of liver injury in sepsis [61]. Also, Huang et al. [62] reported that anti‐histamine blockers may possess promising impacts in ameliorating liver injury induced by intestinal ischemia reperfusion.

However, no studies demonstrated the role of PAF antagonists or antihistamines on CISP‐evoked hepatotoxicity. Thus, these findings allow us to determine whether RUPA, as a dual blocker for PAF and histamine‐1 receptors, would protect against hepatotoxicity evoked by CISP. Consequently, levels of PAF and histamine were analyzed in the hepatic homogenates of rats intoxicated with CISP in the presence or absence of RUPA pretreatment to determine their valuable role in mediating such protection.

The current study indicated that CISP‐injected rats exhibited an increase in hepatic tissue levels of histamine and PAF in comparison with the CTRL rats. PAF is an inducer of NF‐κB activation [63] and its subsequent inflammatory biomarkers [64]. Contrariwise, levels of histamine and PAF showed a decrease in RUPA‐administered rats in comparison with the CISP‐challenged rats. The decline in the level of PAF expression, which is associated with suppression in p‐NF‐κB p65 expression and attenuates the subsequent proinflammatory cytokines, can reinforce RUPA's potential ameliorative effect against CISP‐evoked hepatotoxicity via modulation of PAF.

It is noteworthy to indicate that histamine and TLR4 expression are closely correlated; previous investigations identified that histamine increases TLR4 expression [65, 66]. We postulated that RUPA down‐regulates the expression of TLR4 and consequently suppresses the TLR4 signaling axis due to its capability to block H1 receptors, which is consistent with Sakr et al. [46].

Our study highlights the protective action of RUPA against CISP‐induced hepatotoxicity through the interplay of the proposed signalling pathways. Despite the promising findings, our study has certain limitations. The study did not assess the therapeutic efficacy of RUPA as a post‐treatment drug for CISP‐induced hepatotoxicity. Moreover, the absence of in vitro validation and the limited inspection of other molecular signalling pathways require additional investigation. Thus, properly designed preclinical and clinical studies are needed to further clarify the translational relevance and clinical utility of these results, thus reinforcing the general evidence base.

## 5 Conclusion

Finally, this study revealed that utilizing the dual antagonist of histamine and PAF drug, RUPA, could possess a potential hepatoprotective impact on hepatic injury evoked by CISP administration. This beneficial effect was confirmed with restoration of the different biochemical biomarkers along with the histopathological characters and counteracting the oxidative stress, the inflammatory status, and pyroptosis. Mechanistically, hepatoprotective effect could be achieved via inhibition of the inflammatory HMGB1/TLR4/NF‐κB signaling cascade as well as suppression of the pyroptosis NLRP3/cleaved caspase‐1 signaling pathway. Consequently, RUPA could be regarded as a promising agent against CISP‐evoked hepatotoxicity.

## Author Contributions

**Shaimaa Mohamed Abdelrahman:** data curation; formal analysis; investigation; methodology; visualization; writing – original draft; resources. **Amany Abdlrehim Bekhit:** validation; supervision; writing – review & editing. **Olivia N. Beshay:** conceptualization; data curation; validation; formal analysis; investigation; project administration; methodology; visualization; supervision; writing – review & editing.

## Funding

The authors have nothing to report.

## Ethics Statement

All animal testing experiments comply with the ARRIVE guidelines. Our experimental protocol was reviewed and approved by the Research Ethics Committee for the Care and Use of Laboratory Animals of the Faculty of Pharmacy, Minia University, Egypt (approval code: MPEC (250505)) in accordance with EU Directive 2010/63 for the protection of animals used for scientific purposes.

## Conflicts of Interest

The authors declare no conflicts of interest.

## Declaration of Generative AI Use

The authors used an artificial intelligence (AI) tool (QuillBot) to assist with grammar and spelling checks during the preparation of this manuscript. All AI‐assisted suggestions were reviewed and verified by the authors. AI tool was not used to generate scientific content, analyze data, or interpret results.

## Data Availability

The data that support the findings of this study are available from the corresponding author upon reasonable request.

## References

[1] S. Ghosh , “Cisplatin: The First Metal Based Anticancer Drug,” Bioorganic Chemistry 88 (July 2019): 102925, 10.1016/j.bioorg.2019.102925. 31003078

[2] S. Dasari and P. Bernard Tchounwou , “Cisplatin in Cancer Therapy: Molecular Mechanisms of Action,” European Journal of Pharmacology 740 (October 2014): 364–378, 10.1016/j.ejphar.2014.07.025. 25058905 PMC4146684

[3] M. N. Alshammari , A. H. Alhowail , M. S. Abdel‐Bakky , and M. A. Aldubayan , “Sacubitril/Valsartan Mitigates Cisplatin‐Induced Liver Injury Through Modulation of Oxidative Stress, Caspase‐3 Activity, and RXR‐α Signaling in Experimental Rats,” Frontiers in Pharmacology 17 (February 2026): 1745023, 10.3389/fphar.2026.1745023. 41727541 PMC12917504

[4] E. A. Elmorsy , S. Saber , R. S. Hamad , et al., “Advances in Understanding Cisplatin‐Induced Toxicity: Molecular Mechanisms and Protective Strategies,” European Journal of Pharmaceutical Sciences 203 (December 2024): 106939, 10.1016/j.ejps.2024.106939. 39423903

[5] J. Akhter , J. Khan , M. Baghel , et al., “NLRP3 Inflammasome in Rosmarinic Acid‐Afforded Attenuation of Acute Kidney Injury in Mice,” Scientific Reports 12, no. 1 (January 2022): 1313, 10.1038/s41598-022-04785-z. 35079027 PMC8789898

[6] L. Wang , Z. Dong , Y. Zhang , and L. Peng , “Emerging Roles of High‐Mobility Group Box‐1 in Liver Disease,” Journal of Clinical and Translational Hepatology 12, no. 12 (December 2024): 1043–1056, 10.14218/JCTH.2024.00317. 39649031 PMC11622203

[7] H. Gaskell , X. Ge , and N. Nieto , “High‐Mobility Group Box‐1 and Liver Disease,” Hepatology Communications 2, no. 9 (September 2018): 1005–1020, 10.1002/hep4.1223. 30202816 PMC6128227

[8] M. E. Abdelmageed , M. A. Nader , and M. S. Zaghloul , “Targeting HMGB1/TLR4/NF‐κB Signaling Pathway by Protocatechuic Acid Protects Against L‐Arginine Induced Acute Pancreatitis and Multiple Organs Injury in Rats,” European Journal of Pharmacology 906 (September 2021): 174279, 10.1016/j.ejphar.2021.174279. 34197778

[9] S. Yang , Y. Zou , C. Zhong , Z. Zhou , X. Peng , and C. Tang , “Dual Role of Pyroptosis in Liver Diseases: Mechanisms, Implications, and Therapeutic Perspectives,” Frontiers in Cell and Developmental Biology 13 (January 2025): 1522206, 10.3389/fcell.2025.1522206. 39917567 PMC11798966

[10] L. Zhu , H. Tong , C. Ren , et al., “Inflammation Unleashed: The Role of Pyroptosis in Chronic Liver Diseases,” International Immunopharmacology 141 (November 2024): 113006, 10.1016/j.intimp.2024.113006. 39213865

[11] S. Wei , W. Ma , B. Zhang , and W. Li , “NLRP3 Inflammasome: A Promising Therapeutic Target for Drug‐Induced Toxicity,” Frontiers in Cell and Developmental Biology 9 (April 2021): 634607, 10.3389/fcell.2021.634607. 33912556 PMC8072389

[12] A. D. Grypioti , G. Kostopanagiotou , C. A. Demopoulos , A. Roussos , and M. Mykoniatis , “Platelet Activating Factor (PAF) Antagonism With Ginkgolide B Protects the Liver Against Acute Injury. Importance of Controlling the Receptor of PAF,” Digestive Diseases and Sciences 53, no. 4 (April 2008): 1054–1062, 10.1007/s10620-007-9982-2. 17934819

[13] H. M. Khalaf , S. M. N. A. Hafez , A. M. Abdalla , N. N. Welson , W. Y. Abdelzaher , and F. A. F. Abdelbaky , “Role of Platelet‐Activating Factor and HO‐1 in Mediating the Protective Effect of Rupatadine Against 5‐fluorouracil‐induced Hepatotoxicity in Rats,” Environmental Science and Pollution Research 29, no. 26 (June 2022): 40190–40203, 10.1007/s11356-022-18899-4. 35119631 PMC9120097

[14] V. González‐Núñez , C. Bachert , and J. Mullol , “Rupatadine: Global Safety Evaluation in Allergic Rhinitis and Urticaria,” Expert Opinion on Drug Safety 15, no. 10 (October 2016): 1439–1448, 10.1080/14740338.2016.1221399. 27500993

[15] H. M. Hafez , E. A. Abdel‐Hakeem , and H. Hassanein , “Rupatadine, a Dual Antagonist of Histamine and Platelet‐Activating Factor (Paf), Attenuates Experimentally Induced Diabetic Nephropathy in Rats,” Naunyn‐Schmiedeberg's Archives of Pharmacology 393, no. 8 (August 2020): 1487–1500, 10.1007/s00210-020-01856-8. 32200462

[16] M. A. Didamoony , A. M. Atwa , and L. A. Ahmed , “Modulatory Effect of Rupatadine on Mesenchymal Stem Cell‐Derived Exosomes in Hepatic Fibrosis in Rats: A Potential Role for miR‐200a,” Life Sciences 324 (July 2023): 121710, 10.1016/j.lfs.2023.121710. 37084952

[17] M. Z. Mohamed , H. H. Mohammed , and H. M. Khalaf , “Therapeutic Effect of Rupatadine Against L‐Arginine‐Induced Acute Pancreatitis in Rats: Role of Inflammation,” Canadian Journal of Physiology and Pharmacology 100, no. 2 (February 2022): 176–183, PubMed PMID: 35050802 10.1139/cjpp-2021-0330. 35050802

[18] L. Jiang , Z. Zhang , Z. Luo , et al., “Rupatadine Inhibits Colorectal Cancer Cell Proliferation Through the PIP5K1A/Akt/CDK2 Pathway,” Biomedicine & Pharmacotherapy 176 (July 2024): 116826, 10.1016/j.biopha.2024.116826. 38838507

[19] M. A. Didamoony , A. M. Atwa , and L. A. Ahmed , “A Novel Mechanistic Approach for the Anti‐Fibrotic Potential of Rupatadine in Rat Liver via Amendment of Paf/Nf‐ĸb p65/TGF‐β1 and hedgehog/HIF‐1α/VEGF Trajectories,” Inflammopharmacology 31, no. 2 (April 2023): 845–858, 10.1007/s10787-023-01147-7. 36811777 PMC10140091

[20] X. xi Lv , X. xing Wang , K. Li , et al. Rupatadine Protects against Pulmonary Fibrosis by Attenuating PAF‐Mediated Senescence in Rodents. C. Feghali‐Bostwick , editor. PLoS ONE. 2013 Jul 15;8(7):e68631. 10.1371/journal.pone.0068631. PMC3711902 23869224

[21] T. Bhatia and S. Sharma , “Drug Repurposing: Insights into Current Advances and Future Applications,” Current Medicinal Chemistry 32, no. 3 (January 2025): 468–510, 10.2174/0109298673266470231023110841. 37946344

[22] K. Saranraj and P. U. Kiran , “Drug Repurposing: Clinical Practices and Regulatory Pathways,” Perspectives in Clinical Research 16, no. 2 (April 2025): 61–68, 10.4103/picr.picr_70_24. 40322475 PMC12048090

[23] M. A. Ibrahim , A. A. Abdelmonaem , S. A. Abdel‐Gaber , H. M. Hafez , S. M. N. Abdel Hafez , and W. Yehia Abdelzaher , “Rupatadine Ameliorated Ulcerative Colitis in Rats via Modulation of Platelet‐Activatiweng factor/interleukin‐6/vascular Endothelial Growth Factor Signalling Pathway,” Journal of Pharmacy and Pharmacology 74, no. 4 (April 2022): 537–546, 10.1093/jpp/rgab170. 35134225

[24] F. Shahid , Z. Farooqui , T. Alam , S. Abidi , I. Parwez , and F. Khan , “Thymoquinone Supplementation Ameliorates Cisplatin‐Induced Hepatic Pathophysiology,” Human & Experimental Toxicology 40, no. 10 (October 2021): 1673–1684, 10.1177/09603271211003645. 33832332

[25] N. A. El‐Shitany and B. Eid , “Proanthocyanidin Protects Against Cisplatin‐Induced Oxidative Liver Damage Through Inhibition of Inflammation and NF‐κβ/TLR‐4 Pathway: El‐SHITANY and EID,” Environmental Toxicology 32, no. 7 (July 2017): 1952–1963, 10.1002/tox.22418. 28371137

[26] J. Schindelin , I. Arganda‐Carreras , E. Frise , et al., “Fiji: An Open‐Source Platform for Biological‐Image Analysis,” Nature Methods 9, no. 7 (July 2012): 676–682, 10.1038/nmeth.2019. 22743772 PMC3855844

[27] C. L. Kielkopf , W. Bauer , and I. L. Urbatsch , “Bradford Assay for Determining Protein Concentration,” Cold Spring Harbor Protocols 2020, no. 4 (April 2020): pdb.prot102269, 10.1101/pdb.prot102269. 32238597

[28] N. Abd Rashid , S. A. S. Abd Halim , S. L. Teoh , et al., “The Role of Natural Antioxidants in Cisplatin‐Induced Hepatotoxicity,” Biomedicine & Pharmacotherapy 144 (December 2021): 112328, 10.1016/j.biopha.2021.112328. 34653753

[29] C. A. Oliveira , É. A. B. Mercês , F. S. Portela , et al., “An Integrated View of Cisplatin‐Induced Nephrotoxicity, Hepatotoxicity, and Cardiotoxicity: Characteristics, Common Molecular Mechanisms, and Current Clinical Management,” Clinical and Experimental Nephrology 28, no. 8 (August 2024): 711–727, 10.1007/s10157-024-02490-x. 38678166

[30] B. Yalinbas‐Kaya , A. Tureyen , S. Cesur , F. Zemheri‐Navruz , H. H. Demirel , and S. Ince , “Iristectorin A Ameliorates Cisplatin‐Induced Hepatorenal Injury in Mice Through Modulation of the Nrf2/HO‐1 Signaling Pathway,” Journal of Biochemical and Molecular Toxicology 39, no. 1 (January 2025): e70136, 10.1002/jbt.70136. 39776262

[31] S. M. Satyam , L. K. Bairy , A. Rehman , et al., “Dapagliflozin: A Promising Strategy to Combat Cisplatin‐Induced Hepatotoxicity in Wistar Rats,” Biology 13, no. 9 (August 2024): 672, 10.3390/biology13090672. 39336099 PMC11428795

[32] E. Mansouri , Z. Shafiei Seifabadi , N. Azarbarz , and M. Zare Moaiedi , “Effects of Sodium Hydrosulfide (NaHS) on Cisplatin‐Induced Hepatic and Cardiac Toxicity,” Drug and Chemical Toxicology 47, no. 2 (March 2024): 227–234, 10.1080/01480545.2023.2242008. 37553904

[33] A. Elkomy , E. Y. Abdelhiee , S. E. Fadl , et al., “L‐Carnitine Mitigates Oxidative Stress and Disorganization of Cytoskeleton Intermediate Filaments in Cisplatin‐Induced Hepato‐Renal Toxicity in Rats,” Frontiers in Pharmacology 11 (September 2020): 574441, 10.3389/fphar.2020.574441. 33117167 PMC7552923

[34] S. Kannampuzha and A. V. Gopalakrishnan , “Protective Role of Ursolic Acid Against Cisplatin‐Induced Oxidative Stress and Ferroptosis in the Liver of Swiss Albino Mice,” Medical Oncology 42, no. 8 (July 2025): 327, 10.1007/s12032-025-02854-7. 40652144

[35] N. S. Kalkan and A. Gökçimen , “Protective Structural and Enzymatic Roles of Catechin and Gallic Acid Against Nephrotoxicity and Hepatotoxicity Induced by Cisplatin,” Scientific Reports 16, no. 1 (February 2026): 9046, 10.1038/s41598-025-15445-3. 41690973 PMC12992839

[36] M. Umar Ijaz , A. Ashraf , A. Ahmed , et al., “Remedial Effects of Casticin as An Antioxidant on Cisplatin Induced Oxidative Damage in Rat Liver,” Journal of King Saud University ‐ Science 32, no. 1 (January 2020): 1100–1105, 10.1016/j.jksus.2019.10.009.

[37] I. F. Okkay , A. Famurewa , C. Bayram , et al., “Arbutin Abrogates Cisplatin‐Induced Hepatotoxicity via Upregulating Nrf2/HO‐1 and Suppressing Genotoxicity, NF‐κB/iNOS/TNF‐α and caspase‐3/Bax/Bcl2 Signaling Pathways in Rats,” Toxicology Research 13, no. 3 (May 2024): tfae075, 10.1093/toxres/tfae075. 38770183 PMC11102346

[38] S. Cesur , B. Yalinbas‐Kaya , A. Tureyen , F. Zemheri‐Navruz , H. H. Demirel , and S. Ince , “Obtusifolin Improves Cisplatin‐Induced Hepatonephrotoxicity via the Nrf2/HO‐1 Signaling Pathway,” Naunyn‐Schmiedeberg's Archives of Pharmacology 398 (February 2025): 10337–10352. 10.1007/s00210-025-03900-x. 39976722 PMC12350600

[39] I. O. Sherif , “Hepatoprotective Effect of Arjunolic Acid Against Cisplatin‐Induced Hepatotoxicity: Targeting Oxidative Stress, Inflammation, and Apoptosis,” Journal of Biochemical and Molecular Toxicology 35, no. 4 (April 2021): e22714, 10.1002/jbt.22714. 33491850

[40] A. M. Gendy , M. R. Elnagar , M. M. Allam , et al., “Berberine‐Loaded Nanostructured Lipid Carriers Mitigate Warm Hepatic Ischemia/Reperfusion‐Induced Lesion Through Modulation of HMGB1/TLR4/NF‐κB Signaling and Autophagy,” Biomedicine & Pharmacotherapy 145 (January 2022): 112122, 10.1016/j.biopha.2021.112122. 34489150

[41] S. Y. Alomar , R. E. A. E. Gheit , E. T. Enan , et al., “Novel Mechanism for Memantine in Attenuating Diabetic Neuropathic Pain in Mice via Downregulating the Spinal HMGB1/TRL4/NF‐kB Inflammatory Axis,” Pharmaceuticals 14, no. 4 (April 2021): 307, 10.3390/ph14040307. 33915770 PMC8065430

[42] T. M. Salah , M. A. Rabie , and N. S. El Sayed , “Renoprotective Effect of Berberine in Cisplatin‐Induced Acute Kidney Injury: Role of Klotho and the AMPK/mtor/ULK1/Beclin‐1 Pathway,” Food and Chemical Toxicology 196 (February 2025): 115179, 10.1016/j.fct.2024.115179. 39645019

[43] Q. Man , Y. Deng , P. Li , et al., “Licorice Ameliorates Cisplatin‐Induced Hepatotoxicity Through Antiapoptosis, Antioxidative Stress, Anti‐Inflammation, and Acceleration of Metabolism,” Frontiers in Pharmacology 11 (November 2020): 563750, 10.3389/fphar.2020.563750. 33240085 PMC7683576

[44] H. Hassan , L. Al‐Wahaibi , M. Elmorsy , and Y. Mahran , “Suppression of Cisplatin‐Induced Hepatic Injury in Rats Through Alarmin High‐Mobility Group Box‐1 Pathway by Ganoderma Lucidum: Theoretical and Experimental Study,” Drug Design, Development and Therapy 14 (June 2020): 2335–2353, 10.2147/DDDT.S249093. 32606602 PMC7296982

[45] L. F. L. Malheiro , C. A. Oliveira , F. S. Portela , et al., “High‐Intensity Interval Training Alleviates Liver Inflammation by Regulating the TLR4/NF‐κB Signaling Pathway and M1/M2 Macrophage Balance in Female Rats With Cisplatin Hepatotoxicity,” Biochemical and Biophysical Research Communications 733 (November 2024): 150712, 10.1016/j.bbrc.2024.150712. 39317112

[46] A. G. Sakr , H. E. Kafl , and D. H. El‐Kashef , “Rupatadine Modulates TLR4/MYD88/NF‐κB and AKT/PI3K Signaling Pathways, Attenuating Sepsis‐Induced Liver Injury in Mice,” European Journal of Pharmacology 1002 (September 2025): 177873, 10.1016/j.ejphar.2025.177873. 40562198

[47] N. Abd Rashid , F. Hussan , A. Hamid , et al., “Polygonum Minus Essential Oil Modulates Cisplatin‐Induced Hepatotoxicity Through Inflammatory and Apoptotic Pathways,” EXCLI Journal 2, no. 19 (May 2020): 1246–1265, 10.17179/EXCLI2020-2355. PMC7590832 33122975

[48] H. Abd‐Elgawad , N. Abu‐Elsaad , A. El‐Karef , and T. Ibrahim , “Piceatannol Increases the Expression of Hepatocyte Growth Factor and IL‐10 Thereby Protecting Hepatocytes in Thioacetamide‐Induced Liver Fibrosis,” Canadian Journal of Physiology and Pharmacology 94, no. 7 (July 2016): 779–787, 10.1139/cjpp-2016-0001. 27186801

[49] X. Li , D. Klintman , T. Sato , et al., “Interleukin‐10 Mediates the Protective Effect of Linomide by Reducing CXC Chemokine Production in Endotoxin‐Induced Liver Injury,” British Journal of Pharmacology 143, no. 7 (December 2004): 865–871, 10.1038/sj.bjp.0706015. 15492015 PMC1575945

[50] H. A. Omar , W. R. Mohamed , H. H. Arab , and E. S. A. Arafa. Tangeretin Alleviates Cisplatin‐Induced Acute Hepatic Injury in Rats: Targeting MAPKs and Apoptosis. K. Acharya , editor. PLoS ONE. 2016 Mar 31;11(3):e0151649. 10.1371/journal.pone.0151649. PMC4816535 27031695

[51] M. Z. Mohamed and H. H. Mohammed , “Rupatadine Protects the Intestinal Mucosa From Injury by 5‐flurouracil via Modulation of Inflammation, Apoptosis and Intestinal Permeability,” Drug and Chemical Toxicology 45, no. 6 (November 2022): 2843–2851, 10.1080/01480545.2021.1997541. 34747284

[52] C. Yu , P. Chen , L. Miao , and G. Di , “The Role of the NLRP3 Inflammasome and Programmed Cell Death in Acute Liver Injury,” International Journal of Molecular Sciences 24, no. 4 (February 2023): 3067, 10.3390/ijms24043067. 36834481 PMC9959699

[53] Y. Zhang , M. Yan , W. Shan , et al., “Bisphenol A Induces Pyroptotic Cell Death via ROS/NLRP3/Caspase‐1 Pathway in Osteocytes Mlo‐Y4,” Food and Chemical Toxicology 159 (January 2022): 112772, 10.1016/j.fct.2021.112772. 34929351

[54] H. Chen , S. Wang , Q. Chen , et al. Aloperine Ameliorates Acetaminophen‐Induced Acute Liver Injury through HMGB1/TLR4/NF‐κB and NLRP3/Inflammasome Pathway. F. Tsuji , editor. Mediators of Inflammation. 2024 Jan;2024(1). 10.1155/2024/3938136. PMC11461077 39381066

[55] A. E. D. E. S. El‐Sisi , S. S. Sokar , A. M. Shebl , D. Z. Mohamed , and S. E. S. Abu‐Risha , “Octreotide and Melatonin Alleviate Inflammasome‐Induced Pyroptosis Through Inhibition of TLR4‐NF‐κB‐NLRP3 Pathway in Hepatic Ischemia/Reperfusion Injury,” Toxicology and Applied Pharmacology 410 (January 2021): 115340, 10.1016/j.taap.2020.115340. 33264646

[56] X. Qu , H. Gao , L. Tao , et al., “Autophagy Inhibition‐Enhanced Assembly of the NLRP3 Inflammasome Is Associated With Cisplatin‐Induced Acute Injury to the Liver and Kidneys in Rats,” Journal of Biochemical and Molecular Toxicology 33, no. 1 (January 2019): e22208, 10.1002/jbt.22228. 30291731

[57] D. M. Stafforini , T. M. McIntyre , G. A. Zimmerman , and S. M. Prescott , “Platelet‐Activating Factor, a Pleiotrophic Mediator of Physiological and Pathological Processes,” Critical Reviews in Clinical Laboratory Sciences 40, no. 6 (January 2003): 643–672, 10.1080/714037693. 14708958

[58] O. F. Pavão Dos Santos , M. A. Boim , E. J. G. Barros , E. Pirotzky , P. Braquet , and N. Schor , “Effect of Platelet‐Activating Factor Antagonist BN 52063 on the Nephrotoxicity of Cisplatin,” Lipids 26, no. 12Part2 (December 1991): 1324–1328, 10.1007/BF02536558. 1819725

[59] H. Hamano , Y. Ikeda , M. Goda , et al., “Diphenhydramine may be a Preventive Medicine Against Cisplatin‐Induced Kidney Toxicity,” Kidney International 99, no. 4 (April 2021): 885–899, 10.1016/j.kint.2020.10.041. 33307103

[60] A. A. Shaaban , R. A. Zaghloul , H. E. Kafl , and D. H. El‐Kashef , “Ameliorative Effect of Desloratadine Against Cisplatin‐Induced Renal and Testicular Toxicity in Rats: Attention to TLR4/NLRP3 Inflammasome Signaling Pathway,” Life Sciences 316 (March 2023): 121441, 10.1016/j.lfs.2023.121441. 36709911

[61] M. Hattori , M. Yamazaki , W. Ohashi , et al., “Critical Role of Endogenous Histamine in Promoting End‐Organ Tissue Injury in Sepsis,” Intensive Care Medicine Experimental 4, no. 1 (December 2016): 36, 10.1186/s40635-016-0109-y. 27822777 PMC5099302

[62] P. J. Huang , “Effects of Anti‐Histamine Treatment on Liver Injury Triggered by Small Intestinal Ischemia Reperfusion in Rats,” Chinese Journal of Physiology 57, no. 5 (October 2014): 271–278, 10.4077/CJP.2014.BAC230. 25241987

[63] B. O. Ibe , M. F. Abdallah , A. M. Portugal , and J. U. Raj , “Platelet‐Activating Factor Stimulates Ovine Foetal Pulmonary Vascular Smooth Muscle Cell Proliferation: Role of Nuclear Factor‐Kappa B and Cyclin‐Dependent Kinases,” Cell Proliferation 41, no. 2 (April 2008): 208–229, 10.1111/j.1365-2184.2008.00517.x. 18336468 PMC6496610

[64] J. H. Choi , W. J. Chung , S. J. Han , et al., “Selective Involvement of Reactive Oxygen Intermediates in Platelet‐Activating Factor‐Mediated Activation of Nf‐ΚB,” Inflammation 24, no. 5 (October 2000): 385–398, 10.1023/A:1007068010645. 10921504

[65] J. Talreja , M. H. Kabir , M. B. Filla , D. J. Stechschulte , and K. N. Dileepan , “Histamine Induces Toll‐Like Receptor 2 and 4 Expression in Endothelial Cells and Enhances Sensitivity to Gram‐Positive and Gram‐Negative Bacterial Cell Wall Components,” Immunology 113, no. 2 (October 2004): 224–233, 10.1111/j.1365-2567.2004.01946.x. 15379983 PMC1782560

[66] G. Gutiérrez‐Venegas , S. Cruz‐Arrieta , M. Villeda‐Navarro , and J. A. Méndez‐Mejía , “Histamine Promotes the Expression of Receptors TLR2 and TLR4 and Amplifies Sensitivity to Lipopolysaccharide and Lipoteichoic Acid Treatment in Human Gingival Fibroblasts,” Cell Biology International 35, no. 10 (October 2011): 1009–1017, 10.1042/CBI20100624. 21418040

